# Identity Threat and Coping Among British South Asian Gay Men During the COVID-19 Lockdown

**DOI:** 10.1007/s12119-021-09817-w

**Published:** 2021-02-09

**Authors:** Rusi Jaspal

**Affiliations:** Department of Psychology, Nottingham Trent University, NG1-4FQ Nottingham, UK

**Keywords:** Identity threat, Relationships, Coping, British South Asian gay men, COVID-19

## Abstract

This study focuses on the impact of the COVID-19 lockdown on identity, relationships and psychological wellbeing among British South Asian gay men (BSAGM). Interview data from 15 BSAGM were analyzed using qualitative thematic analysis and identity process theory. The analysis yielded the following three themes: (1) Fear of involuntary disclosure of gay identity; (2) Anxiety about relationships; and (3) Coping and casual sexual encounters. BSAGM who returned to the family home during the lockdown reported fear of involuntary disclosure of their sexual identity, exposure to stigma in relation to their sexuality, and anxiety about their relationships with significant others. In order to cope with the resultant threats to identity, some individuals concealed their sexual identity, which could undermine their sense of identity authenticity, and some reported engaging in casual sexual encounters as a means of coping with identity threat. BSAGM may be at high risk of identity threat during the COVID-19 outbreak with limited access to social and psychological support in relation to their sexuality. This in turn may lead to a reliance on ineffective coping strategies, such as sexual risk-taking.

## Introduction

The novel coronavirus disease (COVID-19) is an infectious disease which is caused by SARS-CoV-2. Following its first clinical observations in Wuhan, China in December 2019, the virus rapidly spread to most major towns and cities in the world. In response to the first outbreak in the United Kingdom, the British government imposed a nationwide lockdown and its social distancing policy on 23 March 2020, initially requiring people to leave their homes only for essential purposes, to refrain from visiting the homes of others, and to maintain a physical distance of at least two meters from people outside of their household (Breakwell et al. 2021). This study was conducted during this initial phase of the outbreak when the United Kingdom went into national lockdown.

There has been a significant impact of social distancing and other aspects of the pandemic on mental health and wellbeing in the general population (Banks and Xu 2020; Marroquín et al. 2020). More specifically, social psychological stressors, such as fear, uncertainty and perceived risk, are having adverse effects on people’s psychological wellbeing (Breakwell and Jaspal 2020; Satici et al. 2020). Although there has been some research into psychological wellbeing during the lockdown among people who identify as lesbian, gay, bisexual and trans (LGBT) (e.g., Anderson and Knee 2020; McKay et al. 2020; Phillips et al. 2020), none has focused specifically on those who also identify as ethnic minorities. British South Asian gay men (BSAGM) are a group vulnerable to stressors associated with their minority (and, in some contexts, stigmatized) ethnic, religious and sexual identities (Jaspal 2012). Drawing on identity process theory and building on existing research into identity and psychological wellbeing in this population, this study examines the experiences of the COVID-19 lockdown among BSAGM, focusing, in particular, on its impact on their identity, relationships with others, and psychological wellbeing.

### A Social Psychological Approach to Identity

This study is informed by Identity Process Theory (IPT) (Breakwell 1986, 2015, 2020), which is an integrative social psychological model of identity construction, threat and coping. Sociological approaches to identity tend to examine the primacy of the social group as a source of self-identity and to focus on the role of societal structure in determining the meanings appended to one’s identity (e.g., Stryker and Burke 2000). Conversely, purely psychological theories habitually focus on the cognitive, affective dimensions of identity with limited attention to social context (e.g., Berzonsky 2011). As a social psychological framework, IPT attempts to bridge these perspectives and, in this study, it is used to explore how individuals manage their sense of identity amid changes unfolding in their social context. In particular, the theory provides a useful heuristic lens for examining how particular events and situations (e.g., the imposition of lockdown) may challenge the process of constructing identity and how these challenges are subsequently managed at both social and psychological levels.

According to IPT, identity is constructed through engagement in two universal processes. The assimilation-accommodation process refers to the absorption of new information in the identity structure, which may be novel events that change how we think about ourselves (e.g., ‘I am in lockdown because I am at risk of COVID-19’), and to the adjustment which takes places in order for this new information to become part of the identity structure (e.g., ‘because I am in lockdown with my family, I may have to change some things about myself’). The evaluation process confers meaning and value upon the contents of identity (e.g., ‘being in lockdown is limiting my self-expression’). Essentially, the processes of assimilation-accommodation and evaluation refer to how we absorb and interpret incoming information which come to form part of our identity.

The two identity processes are guided by various motivational principles, which ‘specify the end states that are desirable for identity’ (Breakwell 1986, p. 24). These include self-esteem, self-efficacy, continuity, distinctiveness and coherence. For instance, exposure to homophobia from significant others during lockdown could challenge one’s self-esteem, while the inability to engage in behaviors perceived to be consistent with one’s sexual identity (e.g., meeting with other gay men) during the lockdown could disrupt one’s sense of continuity. When the identity principles are abrogated—for whatever reason—identity is said to be threatened, which is harmful for psychological wellbeing.

Coping is an important dimension of IPT. In response to identity threat, people strive to restore appropriate levels of the identity principles and, thus, to reduce or eradicate the threat to identity. Breakwell (1986, p. 78) has defined coping strategies as ‘any activity, in thought or deed, which has as its goal the removal or modification of a threat to identity’ and describes their operation at intrapsychic, interpersonal and intergroup levels. For instance, an individual exposed to homophobia may deny his sexual identity (intrapsychic); pass himself off as heterosexual when interacting with others (interpersonal); or derive support from other gay men (intergroup). It is important to note that coping strategies are not activated randomly—as Jaspal (2018) observes, the individual will elect the most appropriate strategy in view of their personality, the social context and indeed the availability of particular coping strategies.

### British South Asian Gay Men

There is now a sizeable literature focusing on the identities, experiences and psychological wellbeing of BSAGM. This research shows that rejection from significant others due to their sexual orientation can be distressing, increasing identity concealment motivation and reducing willingness to come out to others (Bhugra 1997; Jaspal et al. 2019; Yip 2004, 2005). This body of research also suggests that negative religious representations concerning homosexuality contribute to the perceived stigma of one’s sexual orientation (Yip 2007). Although negative representations of homosexuality have been challenged in many religious traditions (e.g., Hunt and Yip 2012; Yip 2005), they are often drawn upon in the attempt to substantiate the widespread view that homosexuality is morally wrong. These beliefs can undermine both identity processes and psychological wellbeing.

There has been research into perceptions and experiences of coming out (Jaspal and Siraj 2011), relationships with others on the gay scene (Bassi 2008) and the cultural expectation of heterosexual marriage (Jaspal 2014). This work suggests that BSAGM are at high risk of identity threat due to exposure to homophobia, racism and cultural pressures to conform to heterosexuality. In an early study of coming out among BSAGM, Bhugra (1997) described feelings of regret, self-deprecation and self-hatred in his participant sample, in view of the ‘traumatic discrepancy’ between their ethnicity and sexual orientation and the psychological difficulties associated with attempting to reconcile these identities. Furthermore, actual and anticipated stigma from significant others and from valued social group memberships, such as one’s family, ethnicity and religion, can undermine one’s sense of self-esteem (Yip 2007). More recently, there has also been research into the identities and psychological wellbeing of British South Asian parents with a son who comes out as gay, which has shown that they too are at risk of identity threat (Jaspal 2020).

It is acknowledged that individuals ‘come out’ as gay to varying degrees (Mohr and Fassinger 2000). One may decide to make one’s sexual identity known to some but not to others. Furthermore, one’s sexual identity may be known by significant others but never discussed openly following the initial coming out event. In response to parental censure of their sexuality, some BSAGM decide to move away from the family home under the pretext of work or study (because it might not otherwise be culturally appropriate to do so, see Beeney 2015). Living away from home enables some to enact their sexual identities relatively freely. Individuals may retain positive relations with their parents and family by ‘compartmentalizing’ their sexual identity from their family identity, that is, the two identities are kept separate. This amounts to a form of coping with anticipated or actual threats to identity, since individuals are attempting to assimilate and accommodate both their sexual and family identities and to ensure their cohabitation in the identity structure by keeping them apart. Yet, as indicated in this study, for some BSAGM, the lockdown has presented challenges for the assimilation-accommodation of these identities and undermined the strategies used to cope with associated threats to identity.

### The Social and Psychological Aspects of Lockdown

Although essential to reduce cases of COVID-19, the lockdown may give rise to additional social and psychological challenges. In the general population, there has been an observed rise in family problems, such as tensions between family members, domestic abuse, including physical and psychological violence, and lack of personal space (Bradbury-Jones and Isham 2020; Mackolil and Makolil 2020; Usher et al. 2020). Furthermore, it has been predicted that the lockdown may lead to the onset of poor mental health outcomes, such as depression, stress and anxiety, and that this may be especially pronounced in vulnerable individuals (Kanter and Manbeck 2020). Indeed, past studies reveal a strong link between social distancing and loneliness and the presence of depressive symptomatology (see Holt-Lunstad et al. 2015 for a review; Santini et al. 2020) and this may be accentuated in the context of COVID-19.

There has also been some research specifically into the impact of COVID-19 on LGBT people. Phillips et al. (2020) argue that LGBT people may be affected disproportionately by the pandemic and that a culturally competent approach to their healthcare will be vital for ensuring that their treatment is equitable (see also Döring 2020). More specifically, Anderson and Knee (2020) note that the social distancing policy in response to COVID-19 has further limited the ability of LGBT people to frequent physical spaces associated with their sexual identity and increased reliance on virtual spaces for deriving a sense of community. Similarly, Brennan et al. (2020) note the loss of LGBT social venues during the lockdown in Canada, as well as decreased access to mental health services for LGBT people who are reliant on them.

Salerno et al. (2020) hypothesize that LGBT individuals, and especially those of ethnic minority background, are at increased risk of adversity in the face of COVID-19 because of their disproportionate experience of inequality, including discrimination, job insecurity, poverty, and poor physical and mental health. Crucially, they note that, due to the COVID-19 outbreak, some LGBT people have been required to return to their family home, where they may not be able to be their authentic selves due to non-disclosure or which may be unaccepting of their sexual identities (Green et al. 2020). The LGBT Foundation (2020) reported that eight per cent of the 555 LGBT respondents reported that they did not feel safe where they were living during the lockdown. These findings are generally consistent with the high rates of parental rejection reported by LGBT people and, especially those of ethnic minority background, which can have negative effects on mental health outcomes (Jaspal et al. 2019).

There has also been some work on sexual health in the context of COVID-19, mainly in gay and bisexual men. Globally, there has been some speculation that the lockdown may have led to a reduction in casual sexual encounters in gay men. In their study of 728 gay and bisexual men in the US, McKay et al. (2020) found that 90 per cent of respondents reported only one or no sexual partner in the last 30 days and that in general participants reported modifying their sexual behavior to reduce their risk of COVID-19. Conversely, in a study of 2361 gay and bisexual men in Portugal and Brazil, Lopes de Sousa et al. (2020) found that 53 per cent of respondents reported engaging in casual sex and that those who had been self-isolating for longer were more likely to report casual sex. Moreover, Maatouk et al. (2020) note that in Lebanon there has been an increase in the uptake of post-exposure prophylaxis (to prevent HIV) during the lockdown, suggesting that individuals are engaging in risk behavior. Consistent with identity process theory, it may be that sexual risk-taking is dependent, at least in part, on psychological wellbeing.

There has been no published research focusing specifically on ethnic minority LGBT people during the COVID-19 outbreak in the United Kingdom. BSAGM may be at particularly high risk of poor social and psychological outcomes, given that many do conceal their sexual identities from their parents and family members and that their parents experience difficulties in accepting a gay son. They may be at increased risk of abuse and violence from family members if their sexual identity is revealed to others. This may increase fear and psychological distress, for instance. Furthermore, in view of the precarious conditions in which some BSAGM form intimate relationships, it may be difficult to safeguard and develop these relationships in a social context which is potentially hostile toward their sexual identity. The present study set out to examine these issues. More specifically, the experiences of the COVID-19 lockdown in a sample of BSAGM who returned to the family home are explored with a focus on the impact for identity and on the strategies enacted to cope.

## Methods

### Participants

Using a snowball sampling strategy, 21 self-identified BSAGM were originally recruited on social media and through introductions made by existing interviewees to participate in a social psychological study of their perceived risk of COVID-19 and the impact on their sexual behavior. During the preliminary analysis, it became clear that much of the discussion in interviews focused on individuals’ experiences of the lockdown and identity threat associated specifically with this aspect of the pandemic. Specifically, the threats were attributed to their return to the family home. It was deemed necessary to produce a report focusing on these specific challenges and, thus, only the transcripts of the 15 participants who had returned to the family home were subjected to analysis.

Ten participants were based in the East Midlands and five in West London. Nine individuals were British Pakistani and six were of Indian heritage. Participants were aged between 19 and 26 years. Ten participants were studying toward, or had received, university-level qualifications, and the remaining five had completed college education. Ten interviewees considered themselves “completely out” (including to their parents); 3 as “out to most people” (but not their parents); and 2 as “out only to close friends.” It is noteworthy that the ten participants who had disclosed their sexual identity to their parents reported that this topic was never discussed and was actively avoided. Six participants reported being in a romantic relationship and 11 were reportedly single. Of the six individuals who were in a relationship, five indicated that they experienced difficulties in disclosing their relationship to other people. All of the participants were living away from their parents but had returned to their parental home during lockdown.

### Analytic Approach

The interviews were guided by a semi-structured interview schedule which included a series of exploratory, open-ended questions concerning self-description and identity, followed by questions/probes focusing on how identity (and especially one’s sexuality) had changed as a result of the COVID-19 outbreak, how individuals perceived the future, and how their relationships with others (especially other gay men and their families) were evolving. Interviews lasted between 30 and 75 minutes and were digitally recorded and transcribed verbatim.

Qualitative thematic analysis, which has been described as ‘a method for identifying, analyzing and reporting patterns (themes) within data’ (Braun and Clarke 2006, p. 78), was used to analyze the data. When used in conjunction with identity process theory, it enables the analyst to examine how aspects of one’s experience have impacted on the construction and management of identity. In this study, thematic analysis was used to identify themes in the interviews concerning the perceived impact of the lockdown on one’s identity, relationships and psychological wellbeing. A realist epistemological stance was employed and, accordingly, participants’ accounts in interviews were considered to reflect underlying cognitions and emotions.

The author transcribed the interview recordings and conducted a preliminary analysis of the transcripts. During each reading of the transcripts, initial interpretations were noted on each transcript. The emerging codes were then collated into potential themes, which captured the essential qualities of the accounts. The list of themes was reviewed rigorously against the data to ensure their compatibility and extracts were listed against each corresponding theme. At this stage interview extracts, which were considered representative of the themes, were selected for presentation in this article. Finally, three superordinate themes that reflected the analysis were developed and ordered into a coherent narrative structure.

## Results

In this section, the following three themes are described: (1) Fear of involuntary disclosure of gay identity; (2) Anxiety about relationships; and (3) Coping and casual sexual encounters.

### Fear of Involuntary Disclosure of Gay Identity

Several interviewees outlined the difficult decision to return to their family home, rather than stay away, when the lockdown was announced. They described feeling compelled to ‘choose between’ their sexual and family identities, which they ordinarily compartmentalized to avoid threats to psychological coherence.

It was like ‘do I stay here and basically just end up alone but being myself or do I go back there and stay with my family but go right back in the closet?’ It’s a really tough call. I went back and it’s got its good points too but I do find myself being more anxious about stuff, you know, overthinking stuff. (Baljit)

During the lockdown I’m facing quite a bit of my inner demons, and I knew I would at home, so you know I was in two minds about going back… Now I’m just thinking about my future choices and coming out and this and that. (Imran)

Although ten participants described themselves as being ‘completely out’, all of them acknowledged difficulties in discussing their sexual identities (and particularly romantic relationships) with their parents and other family members. Their parents were aware of their sexual identities but this was never openly discussed. Individuals had developed ways of enacting their sexual identity away from the family home—for instance, they had developed social networks consisting of gay friends.

Yet, the prospect of returning to the family home posed a potential threat to their sense of continuity because the interviewees were aware that it would be impossible to continue to enact their sexual identity in quite the same way. For example, they anticipated challenges in retaining their gay social network during lockdown. Furthermore, there was evidence that some individuals had been protecting identity (and especially their sense of continuity) by avoiding acknowledgement of their parents’ difficulties with their sexual identity:

I just pushed it [parents’ reaction to his sexual identity] right the way to the back of my mind and just sort of like wished it away. That’s the only way I could just get on with my life. (laughs) (Sukhy).

Put simply, living away from the family home enabled them to avoid thinking about their parents’ (potential) stance on their sexual identity while returning home led them to ruminate about it. Thus, the prospect of lockdown increased the risk of discontinuity in identity since interviewees felt compelled to acknowledge these difficulties and to ‘face their inner demons’. Being in lockdown forced some to confront issues that they had previously deflected or denied, which in turn increased negative affect.

One of the issues that caused negative affect was participants’ concern about the heightened risk of involuntary disclosure of their sexual identity during lockdown. More specifically, there was a perception that the measures previously developed to avoid involuntary disclosure were being destabilized during the lockdown:

When I’m at home, it’s harder to, you know, talk on the phone and keep in touch and when I call, I’m whispering. His [boyfriend’s] family are there and he’s got the same problem. I’m just scared of it all blowing up. (Ahmed)

My parents know I’m gay but it isn’t really talked about. We don’t talk about it…. it’s more hard now because more time for this to like come up. They ask me stuff and it’s just hard. It makes me feel bad about myself, I don’t know why. (Manjit)

Our parents never really see us as grown-ups so my mum sees the right to just say ‘who was that you were talking to?’ and so that becomes a bit of a debate. (Reza)

Participants were reticent about involuntary disclosure of their sexual identity and even those individuals who had disclosed their identity to their families were generally careful to avoid raising the topic. Some described their sexual identity as a ‘no go area’ because it was never discussed and, in some cases, because it could cause tensions among family members who preferred not to acknowledge this. Ahmed described his fear that his conversation with his boyfriend might be overheard which could lead to the issue of his sexual identity ‘all blowing up’. Incidentally, he reported that his boyfriend faced the same challenge at home and was also fearful of involuntary disclosure of his identity.

Similarly, Reza described the discontinuity in identity engendered by his decreased sense of autonomy at home. More generally, several interviewees noted that the lockdown had imposed discontinuity in their daily routine, which in turn provided scope and opportunity for family members to discuss issues that might ordinarily be avoided, deflected and denied. This sense of ‘structural discontinuity’ (that is, in their daily routine) also created discontinuity in identity since some individuals feared the future.

As Manjit noted, this was challenging not only for their sense of continuity, which was safeguarded by avoiding overt acknowledgement of their sexuality, but also self-esteem. There was an element of internalized homophobia among some individuals who had not entirely resolved threats to identity associated with being gay—indeed, exposure to stigmatizing remarks from significant others, such as their parents, increased the risk of threatened self-esteem. In concealing, and avoiding discussion of, their sexual identity, individuals were able to protect their self-esteem. However, there were concerns that this strategy of non-disclosure/ non-acknowledgement could be undermined during lockdown, essentially forcing them to acknowledge a stigmatized element of identity, which they would usually attenuate.

There was a pervasive desire to conceal sexual identity which helped avert threats to self-esteem and continuity associated with sexual identity disclosure. Yet, in attempting to protect these identity principles, individuals also described a consequential loss of identity authenticity:

It’s a really weird feeling because I’m hiding who I am and it feels a bit like I’m not the real me so it’s hard being here sometimes. (Karan)

At home I’m playing the straight guy role because that’s what they expect of me, my parents. (Kam)

The coping strategy of identity concealment was deemed to reduce threats to self-esteem and continuity because participants were not exposed to stigmatizing remarks or actions from significant others. The risk that exposure to such remarks and actions might reactivate internalized homophobia was similarly reduced. Yet, in utilizing the strategy of identity concealment, some interviewees felt unable to derive a sense of identity authenticity since they were aware that, by concealing their identity, they were not being ‘the real me’. They were simply feigning heterosexuality in order to conform to their parents’ expectations. They had replaced the authentic sense of self developed prior to the lockdown with an inauthentic sense of self consistent with others’ expectations.

### Anxiety about Relationships

As indicated in the previous section, maintaining intimate relationships represented a key challenge during the lockdown. Individuals described the need to conceal their sexual identity from their families, which reportedly had a negative impact on the quality of their romantic relationships. Some interviewees experienced a feeling of estrangement from their intimate partners:

I get a bit worried sometimes because it’s, we [my boyfriend and I] don’t have friends in common so it was quite intense before and now I’m here, he’s there. I’m here physically but there mentally and my family get that. They see that I’m not totally here. (Baljit)

It’s hard to call and speak so I’m a bit worried because we don’t talk as often and before I was seeing him a lot and it could just, he could be cheating on me or maybe find someone else. (Ahmed)

In previous research, it has been shown that BSAGM experience challenges in developing and maintaining romantic relationships and in managing relationship dissolution because many pursue their relationships in secrecy and derive only limited social support from others (Jaspal 2015). Similarly, Baljit described the insularity and intensity of his relationship with his partner—more specifically, he reported that they did not have friends in common and that they were heavily reliant on one another. For both Baljit and Ahmed, the lockdown exerted significant pressure on their respective relationships because, on the one hand, they were unable to communicate as frequently and effectively as they could before the lockdown and, on the other hand, they had no friends in common who might ordinarily provide a sense of connection with their relationship. This abrupt change to their relationships clearly represented a threat to continuity—they were compelled to accept a change in their relationship which was unanticipated and undesirable and for which they were ill-prepared. Some interviewees felt anxious about the future of their relationship, fearing that their partners might be unfaithful or ‘find someone else’. Baljit, in particular, reported a sense of dislocation between his physical and psychological being.

Participants expressed anxiety about the future of their relationship because of the impact of the lockdown:

When I’m in London, I ring my parents and we chat and it’s all fine. Here I’m arguing with them asking me when I’m going to settle down [with a woman] and then I end up taking it out on him [my boyfriend] when I call him. He’s had enough. I’ve had enough. (Raj)

We’re all in this corona situation together. A lot of people are having a hard time but I think it’s harder when you’ve got a secret relationship like me and you can’t get out, do what you used to do. It’s just that bit harder for us so it makes me nervous about the future. (Baljit)

Interviewees appeared to be experiencing a threat to continuity because of the adverse changes that were occurring in their lives. During the lockdown, several were confronted by their parents about their sexuality and questioned about their marital intentions, that is, when they would get married (to a woman) and whether this would be an arranged marriage. For Baljit, this added a further layer of complexity to his relationship with his partner, as he felt less able to communicate with him, fearing that their conversation might be overheard by his parents and family members. Raj acknowledged his low mood in the few interactions he had with his partner. In view of this adverse impact on relationship quality, the threat to continuity consisted of a rupture not only between past and present but also between present and future, which was perceived to be uncertain. In short, interviewees did not know what to expect about their relationships in the future.

Lockdown reportedly provided some individuals with the opportunity to reflect on their identities and, more specifically, on the role of their intimate relationships within their identity. Having previously been suppressed, the threat to psychological coherence in relation to their intimate relationships and family identity could now gain access to consciousness:

I’ve had a lot of time to think and it makes me feel like I’m going to have a panic attack... I’ve been ignoring the future for a long time but now it’s like there, in your face. I don’t want to be leading a double life. (Ali)

It’s brought home to me you know that I’ve got to choose basically. My parents aren’t going to accept this relationship and I know I’ve got to keep my family at arm’s length if I’m going to be happy with my life. Being at home has basically shown this. (Salim)

Across the participant sample, there was an underlying threat to psychological coherence in that two significant components of identity were deemed to be incompatible—their family identity and sexual identity. Even those ten individuals who had disclosed their sexual identity to their parents reported that the topic was never discussed and actively avoided, suggesting that this still constituted a challenge for their relationship with their parents (Jaspal 2020). This threat to psychological coherence was never really resolved in its entirety but rather deflected from identity in that individuals attempted to avoid thinking about it. However, as indicated in the accounts above, the lockdown compelled individuals to confront this threat once again, which induced negative emotional experiences. While living away from their parents, individuals were able to suppress the threat to psychological coherence. However, during the lockdown, the threat re-surfaced and simply could not be denied. The threat consisted of the widespread perception among interviewees that their sexuality would never fully be understood or accepted by their parents, leaving the two components of identity (family vs. sexuality) polarized in the identity structure. Crucially, individuals did wish to retain both identity components but believed that this was impossible. In the interviews, there was little evidence of any adaptive, long-term strategy for resolving this threat to psychological coherence.

### Coping and Casual Sexual Encounters

During the lockdown, individuals reported exposure to multiple stressors, including stigma, fear of involuntary disclosure of their sexual identity and relationship problems. Several interviewees reported engaging in casual sexual encounters in order to minimize the impact of the multiple stressors that they experienced during this period:

When we were all locked indoors, I was like pulling my hair out, thinking that I can’t take this for much longer. Especially trapped in with my dad watching me like a hawk. Literally the only way of meeting anyone from outside – like proper meeting – was on Grindr. (Saj)

Waqar: For me, it was just an escape really.

Interviewer: From what?

Waqar: Being stuck at home when I’m not used to it. Parents stressing me out. Boyfriend stressing me out on the phone. Having sex felt good.

They [the government] were saying ‘no contact’ but you know there’s always someone about online up for meeting. (Aaron)

Some interviewees reported that they felt that they were being supervised by their parents, which contrasted negatively with their more favorable experience of enacting their sexual identity before the lockdown. Individuals reported using geospatial gay social networking applications, such as Grindr, in order to meet for casual sex with other gay men. As Aaron suggested, despite the coercive norm of social distancing, online applications provided some psychological respite in that they facilitated face-to-face encounters with other gay men. There was a strong sense that casual sex constituted a form of escapism from the threatening experiences that individuals were facing during lockdown at home, such as being watched, having their behavior regulated by parents and family members, and being ‘locked indoors’. In reflecting on the psychological functions performed by their casual sexual encounters, participants generally described them as enabling them to cope with these negative aspects of the lockdown. As a form of escapism, casual sexual encounters enabled individuals to disconnect from these threatening experiences during this challenging period:

I just felt this really big release. Like I got something totally off my shoulders, off my back, and it felt really good. (Ali)

To be honest with you, I love my family, yeah, but you know I feel this massive distance because of all the lies and all the hiding. It’s like I’m there but a million miles away... Finally just with some random guy, you know, there was like this closeness between him and me during that moment and that felt good. (Sukhy)

I just did it for some intimacy. I’m used to that [meeting for casual sexual encounters] in London and back home I wasn’t and it was like ‘what is going on with my life?’ It just felt like I wasn’t doing what I usually do, log onto Grindr and meet up. (Kam)

It is important to note that none of the participants had access to support networks consisting of gay men during the lockdown—due to a lack of privacy at home, they were unable to connect satisfactorily with the gay social networks that they had constructed elsewhere. Interviewees described relatively superficial relations with their parents, which were devoid of emotional intimacy, partly because they felt that they were having to be inauthentic in order to avoid parental censure of their sexualities. Sukhy’s account suggested that the emotional ‘distance’ that characterized his relationship with his parents made him long for a sense of intimacy with others.

Some derived a sense of continuity between their pre-lockdown lifestyle and their current position during lockdown by meeting with gay men on Grindr and other applications. Despite the potential risks associated with meeting with others for casual sex (most notably, exposure to COVID-19), interviewees generally constructed a sense of continuity by doing so—this enabled some to feel re-connected with a past that they longed for since the outbreak. This constituted a means of re-establishing a previous lifestyle (characterized by the ability to seek casual sexual encounters and to derive intimacy with other gay men) in a context in which this lifestyle was now stigmatized and even prohibited.

Some individuals acknowledged engaging in sexual risk behaviors during their casual encounters but noted that they did not feel able to seek support after these encounters due to the stigma associated with non-adherence to the social distancing norm:

You know what it’s like. You talk to everyone and they’re all at home, aren’t they? Nobody’s breaking the lockdown but you apparently. It made me feel silly actually. (Mo)

To be honest, I was a bit worried about the police and that, because they were talking about fines on the spot, and maybe it goes on my record so I just kept quiet about it. (Reza)

I did have sex without a condom one time but I didn’t get tested because they’re going to ask me what I was doing having sex in the first place, during lockdown. It’s embarrassing. (Aaron)

Waqar: I have used PEP [post exposure prophylaxis to prevent HIV infection] once before but I didn’t this time even though I did have an accident once. I mean, I didn’t use one [a condom].

Interviewer: Why not?

Waqar: Everything was a mess and I didn’t really know if it was on. It just wasn’t practical.

Individuals were acutely aware of the stigma surrounding non-adherence to the social distancing policy. They reported various negative emotions in relation to their own non-adherence, largely because they came to believe that they were alone in failing to adhere to the policy. They felt excessively distinctive in doing so. Furthermore, there was uncertainty about the legal implications of non-adherence to the policy and some interviewees reported concerns that they might be prosecuted for meeting with people from outside their household. This was especially distressing for those individuals who were also fearful of involuntary disclosure of their sexual orientation. Given that most individuals sought casual sexual encounters late at night, there was some discussion about how they might explain their reason for being outdoors if questioned by a police officer. In other words, the very act of seeking casual sexual encounters posed personal risks. The possible coping strategy of seeking intimacy with other gay men was perceived as posing a risk to their identity and, in some cases, safety.

A small number of interviewees also noted the risks to their sexual health during lockdown. In some cases, the stigma surrounding non-adherence to social distancing and the fear of involuntary sexual identity disclosure impeded access to sexual health screening and also to the uptake of post-exposure prophylaxis to prevent HIV infection after possible exposure to the virus. Although some interviewees reported taking health risks (both in terms of COVID-19 exposure and the acquisition of sexually transmitted infections), they did not generally seek clinical intervention to reduce these risks because of stigma and fear. This demonstrated the insidious effect of stigma on health and wellbeing among BSAGM struggling to cope with identity threat.

## Discussion

Although there is evidence that marginalized minority groups are at especially high risk of poor psychological health outcomes during COVID-19 and the associated lockdown to curb its spread (Kanter and Manbeck 2020), there has been limited research into the dynamics of LGBT people’s lives and, until this study, no research into the impact for BSAGM. This study demonstrates some of the psychological challenges experienced by people from this population as they struggle to construct, reconcile and protect two significant components of their identity—sexuality and family.

### Threats to Identity

It has been reported elsewhere that BSAGM may experience difficulties in reconciling their sexuality, ethnicity and religion and that, in particular, they may compartmentalize their sexual and family identities in order to protect identity (Bhugra 1997; Yip 2007). Indeed, interviewees described constructing a positive sexual identity which was characterized by authenticity while living away from home. More specifically, some participants had been able to engage with LGBT affirmative social contexts, to build friendships with other gay men and to derive a mechanism for compartmentalizing their sexual and family identities. Family identity is important—much research shows that family support, acceptance and knowledge shape the identity adjustment and functioning of gay men (Elizur and Ziv 2001; Pastrana 2015).

In this study, interviewees attempted to achieve a positive identity primarily by deflecting threats to their identity associated with the stigma appended to their sexual identity, by concealing their sexual identity from family members and by compartmentalizing identity elements that might cause tensions and disruption in the identity structure. Yet, these deflection strategies for protecting identity were challenged by their return to their family home during lockdown (Green et al. 2020). For some, this induced a sense of discontinuity in both their daily routine and in identity; threats to self-esteem associated with unresolved internalized homophobia and potential exposure to homophobic stigma from others; and disruption to psychological coherence associated with the perceived incompatibility of their sexual and family identities. Although several individuals reported having disclosed their sexual identity to their parents and family members, there was never any overt acknowledgement of it and, in some cases, the information was denied and deflected by family members (see Mohr and Fassinger 2000), possibly because it was threatening for them (Jaspal 2020). The strategies of deflection, identity concealment and compartmentalization became unsustainable during lockdown for at least two reasons.

First, some interviewees found that the topic of their sexuality (and peripherally related issues, such as having an arranged heterosexual marriage) was reignited by family members during lockdown, thereby forcing them to acknowledge threats to identity and to take a stance on them. While living away from home, BSAGM may derive social support from like-minded others who can provide access to positive social representations of being gay, which in turn can provide psychological respite from the stigmatizing social representations of their sexuality habitually encountered in religious, ethnic and cultural settings (Doty et al. 2010). They may be able to ‘switch off’ these stigmatizing representations by immersing themselves in LGBT-affirmative social settings, thereby reducing the salience of internalized homophobia and protecting self-esteem from potential threats. This does not mean that these negative tenets of identity are completely resolved but rather that they are transiently suppressed. Yet, their return to their family home forced some interviewees to confront and ruminate about the perceived inconsistencies between being gay and British South Asian (and the subcategories included within this broad identity, such as being Muslim, Hindu or Sikh). In short, at a psychological level, they had to take a stance on the threatening aspects of identity, which, in the absence of social support, was difficult.

Second, it became increasingly challenging for some people to continue to conceal their sexuality from family members due to a lack of privacy in the family home. Some believed that they were having to choose between their identity elements—either abstaining from any activity associated with their sexuality (such as communicating with gay friends or intimate partners) in order to avoid family censure of their sexuality or continuing to enact their sexuality and risking threats to their family identity as a consequence. The dilemma associated with sexual identity concealment (designed to protect self-esteem and psychological coherence) and sexual identity enactment (intended to protect identity authenticity and the derivation of social support) was distressing for some interviewees.

Identity authenticity refers to ‘the sense that one’s life, both public and private, reflects one’s real self’ (George 1998, p. 134) and, thus, constitutes an important tenet of identity. While compartmentalization can facilitate identity authenticity since one can at least derive a sense that one is authentic in *some* social contexts, identity concealment in a social context in which one is ‘locked down’ for a prolonged period of time can understandably undermine identity authenticity, potentially exposing BSAGM to identity threat. There were also concerns about involuntary disclosure of their sexual identity, which might cause conflict and tension within the family unit (Green et al. 2020). In order to limit the risk of involuntary identity disclosure, some individuals strategically opted to reduce their level of contact with their gay social networks (developed before the lockdown), thereby imperiling their intimate relationships. They expressed anxiety about the continuity of these relationships, which in turn produced negative affect.

### Coping

Identity process theory outlines a series of coping strategies designed to minimize or eradicate threats to identity (Breakwell 1986). People select coping strategies that are most appropriate to both personality and social context, bearing in mind the availability of particular coping strategies in a given social context. In order to cope with identity threat, negative affect and a perceived lack of intimacy, several interviewees reported seeking sexual encounters with other gay men during lockdown using geo-spatial gay social networking applications, such as Grindr. Some reported engaging in sexual risk behaviors, such as condomless sex, without taking precautions to reduce the risk of HIV and other sexually transmitted infections. In view of the coercive norm of social distancing and the avoidance of contact with people outside of one’s household during the lockdown (Michie et al. 2020), individuals who engaged in sexual risk behaviors were reluctant to seek support from others (as an alternative coping strategy) and to engage with sexual health services (such as HIV testing, sexual health screening and the acquisition of post-exposure prophylaxis to prevent HIV). This reluctance to engage with services must be understood in the broader context of stigma, whereby gay and bisexual men who engage in condomless sex are often positioned as reckless, carefree and impulsive (Jaspal 2018). It must also be noted that the desire to conceal their sexual identity from significant others (to protect identity from other threats) constituted an additional layer of complexity.

## Conclusions

The COVID-19 lockdown has shown the unsustainability and short-lived efficacy of deflection strategies operating at intrapsychic, interpersonal and intergroup levels of coping for people facing identity threat. During the lockdown, some BSAGM are unable to deny and forget, and to isolate themselves and to engage in passing. They may similarly be unable to immerse themselves in social groups that might provide access to positive social representations of their sexuality and to avoid those social groups that might censure their sexuality. They may find themselves in a situation in which it is difficult to derive identity authenticity. As the pandemic has progressed, further local and national lockdowns have subsequently been imposed. As we attempt to understand the impact of the COVID-19 pandemic on psychological wellbeing, it will be necessary to examine the lives and identities of marginalized minority groups with decreased access to social support. BSAGM who return to the family home constitute one such group. This qualitative study of BSAGM’s experiences of the COVID-19 lockdown demonstrates just some of the social, psychological and sexual health challenges faced by individuals from marginalized minority groups. It will important to increase visibility of BSAGM in our efforts to combat poor psychological wellbeing during the pandemic, to tailor social and psychological support to them and, above all, to ensure that the social context in which they seek this support is affirmative of both their sexual and cultural identities.
